# Integrating spore trapping technology with loop-mediated isothermal amplification assay for surveillance and sustainable management of rice false smut disease

**DOI:** 10.3389/fmicb.2024.1485275

**Published:** 2024-12-04

**Authors:** Meena Arumugam Gopalakrishnan, Gopalakrishnan Chellappan, Kamalakannan Ayyanar, Jagadeeswaran Ramasamy, Patil Santhosh Ganapati, Sathyamoorthy Nagaranai Karuppasamy

**Affiliations:** ^1^Department of Plant Pathology, Tamil Nadu Agricultural University, Coimbatore, India; ^2^Department of RS and GIS, Tamil Nadu Agricultural University, Coimbatore, India; ^3^Agricultural Statistics, Tamil Nadu Agricultural University, Coimbatore, India; ^4^Agro Climate Research Centre (ACRC), Tamil Nadu Agricultural University, Coimbatore, India

**Keywords:** spore trap, false smut, LAMP, rice, PCR, *Ustilaginoidea virens*

## Abstract

Rice (*Oryza sativa* L.) is a vital crop feeding more than half of the world’s population, with production occurring predominantly in Asian countries. However, rice cultivation faces challenges from various fronts, including biotic stresses intensified by climate change. False smut, caused by *Ustilaginoidea virens*, has emerged as a significant threat to rice production globally. The application of curative fungicides after symptom appearance has limited scope in managing this disease since the infection process usually starts during the early flowering stage of rice crops. This study investigates the utilization of spore-trapping technology coupled with Loop-Mediated Isothermal Amplification (LAMP) assay for monitoring airborne *U. virens* inocula in rice fields. For early detection and quantification of *U. virens*, sampling rods coated with silicone grease were deployed to collect airborne spores, and DNA extraction was performed using a modified method. Both PCR and LAMP assays were employed for detection, with LAMP offering advantages of rapidity, sensitivity, and simplicity. The study demonstrated the superior sensitivity of LAMP compared to PCR, detecting *U. virens* DNA at concentrations as low as 100 femtograms. Continuous monitoring of *U. virens* inoculum using spore trapping revealed the spatio-temporal dynamics of *U. virens* dispersal, providing valuable insights for disease management. Implementing a fungicidal application schedule based on airborne inoculum detection led to significant reductions in both false smut incidence and severity and improved crop yield. The meteorological parameters including minimum temperature, relative humidity in the morning and evening, sunshine, and solar radiation were found to be correlated with disease incidence. Multi-operator validation confirmed the robustness and specificity of the LAMP assay. Overall, this integrated approach offers a proactive strategy for monitoring and managing false smut disease, enhancing sustainable rice production and food security.

## Introduction

Rice (*Oryza sativa* L.) is prominent in global agriculture and feeds more than half the world’s population. Rice is grown in more than 100 countries, with the majority of production in Asian countries. The USA is also a significant producer of rice, with an estimated production of 11 million metric tons in 2022 (USDA, 2023). However, rice production is hampered by various factors, especially the biotic stress related to climate change. It has been observed that old, historically minor diseases are emerging with high vigor due to climate change. False smut was first reported in India in 1878, and currently, it is prevailing in 6 continents and 59 countries, including Asia, tropical Africa, Australia, Oceania, Europe, and America (Khanal et al.,2023; Dangi et al.,2020). Rice false smut spreads through airborne spores and infects the crop mainly during the booting stage, where individual grain transforms into velvety spores or yellow smut balls. It decreases the grain quality and increases chaffy grain production. False smut not only leads to yield loss but also produces toxins inhibiting/causing abnormal cell mitosis in humans and animals (Yang et al., 2022). After noticing initial symptoms, curative sprays of fungicides were ineffective against rice false smut since the infection process occurs during the early flowering stage (Narinder and Singh, 1989; Thurston, 1990). Hence, the effective management of this pathogen requires a thorough understanding of its dispersal dynamics, particularly the airborne inocula that serve as primary sources of infection. Traditional methods of disease monitoring often lack the temporal and spatial resolution needed to track *U. virens* dispersal accurately. However, recent advancements in spore trapping technology offer a promising avenue for studying the airborne dispersal of fungal pathogens in agricultural environments.

Although spore trap samples could be analyzed using a simple microscope, they are laborious and require a skilled professional (Gowrisri et al., 2019). To overcome these issues, different molecular techniques, such as polymerase chain amplification (PCR), can be used based on reliability and specificity for the detection and quantification of *U. virens*. Though PCR is considered one of the standard and reliable detection methods, it requires a thermal cycler, and the amplification was further confirmed only by gel electrophoresis, apart from the presence of PCR inhibitors in plant samples. Alternatively, the Loop-Mediated Isothermal Amplification (LAMP) assay developed by Notomi et al. (2000) was found to be an alternative to PCR as it works under isothermal conditions, making it ideal for onsite/infield applications. LAMP uses three sets of primers. Hence, they are highly specific and sensitive techniques and visible detection can be made in a short period (30–60 min; Logeshwari et al., 2022). Application of LAMP can be extended for early detection of plant pathogens such as fungi, bacteria, viruses, and phytoplasma (Prasannakumar et al.,2021; Azizi et al., 2022; Rafiq et al., 2021). The present study attempted to utilize a spore trapping technique coupled with the LAMP assay to track and quantify the airborne inocula of *U. virens* with the ultimate goal of devising targeted and effective disease management strategies.

## Materials and methods

### Spore trap and sampling rod preparation

**Figure 1:**
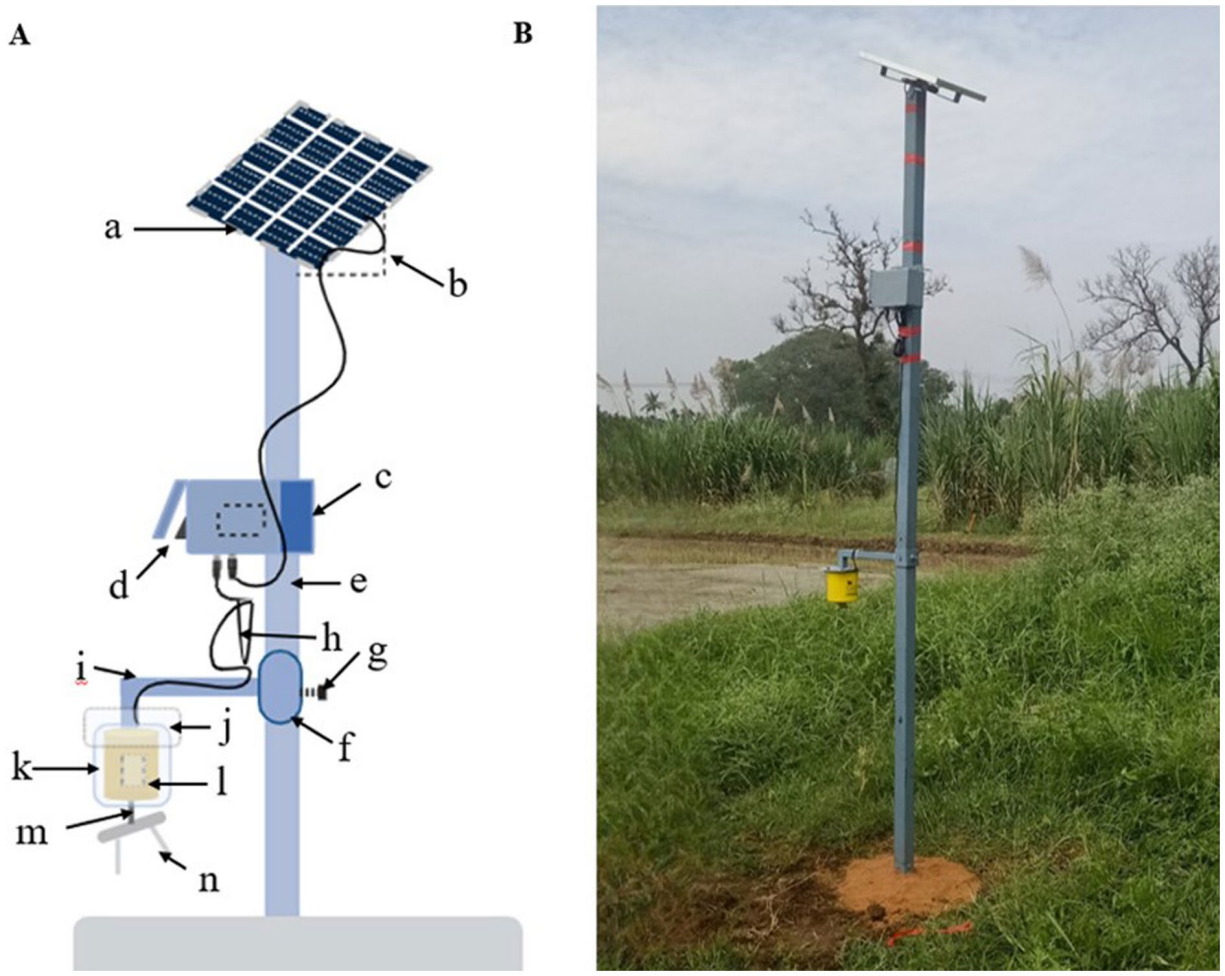
Solar power operated impaction spore trap. **(A)** Layout of impaction spore trap. a: Solar panel (18V); b: Tilt angular cum panel holder; c: Battery (6V); d: On/Off switch; e: G.I pipe; f: Coupling; g: Height adjuster screw; h: Wire (connecting solar panel—battery-motor); i: L rod (motor holder); j: iron plate attached to L rod; k: Plastic causing; l: DC motor (12 V); m: Sampling arm; n: Sampling rods. **(B)** A solar operated impaction spore trap deployed at Paddy Breeding Station, Tamil Nadu Agricultural University, Coimbatore, Tamil Nadu.

This study used a modified low-cost impaction spore trap designed by Chandrasekar (2022). The trap consists of a photovoltaic module, 12 V DC, 300 RPM gear motor, 6,000 mAh Li-ion battery, solar panel, metallic sampling rods, rotating arm, and steel stand (Figure 1).

Sampling rods designed for capturing airborne particles were crafted from 60-mm long and 3 mm-wide stainless steel, following the method outlined by Thiessen et al. (2018) with slight modifications. These rods were sterilized and coated with silicone vacuum grease, and then fixed to the rotary arm of the spore trap for sampling.

### A spore trap was set up in the field

The trap was set up at Paddy Breeding Station, Coimbatore, Tamil Nadu, India (Latitude 11.0068 °N, Longitude 79.9242 °E) with an average rainfall of 670 mm per year. Geographically, it is located at an elevation of 426.72 m, with an average annual minimum temperature of 22°C and a maximum temperature of 33°C for the year 2021 (Indian Meteorological Department official website, 2021, https://mausam.imd.gov.in/imd). The paddy variety Co-39, which is susceptible to false smut, was grown in the nursery, and the 20-day-old seedlings were transplanted in the main field with a spacing of 20 × 15 cm. The spore trap was operated continuously from transplanting to the harvesting stage of rice crops from the 45th Standard meteorological week of 2023 to the 6^th^ Standard meteorological week of 2024. Sampling rods were collected aseptically from the trap using sterile containers on a weekly basis, with two rods collected each week and replaced with sterilized rods freshly coated with grease.

### Microscopic observation

The temporary mounts were prepared by gentle scraping of airborne inocula adhered to the grease of sampling rods collected from the spore trap fixed under field conditions and observed under 40x magnification in a phase contrast microscope for observing false smut conidia. The presence and absence of inocula are mentioned by the + or − sign (Table 1).

**Table 1:** Detection of *U. virens* using spore trap.

S. No	SMW	Microscopic observation	PCR	LAMP assay	PDI calculated for the treatments (%)		
					T_2_	T_1_	T_3_
1.	45^th^	−	−	−	0	0	0
2.	46^th^	−	−	−	0	0	0
3.	47^th^	−	−	−	0	0	0
4.	48^th^	+	−	+	0	0	0
5.	49^th^	+	−	+	0	0	0
6.	50^th^	+	+	+	0	0	0
7.	51^st^	+	+	+	0	6.53	7.64
8.	52^nd^	+	+	+	0	8.24	12.59
9.	1^st^	+	+	+	0	10.36	13.34
10.	2^nd^	+	+	+	0	16.25	17.19
11.	3^rd^	+	+	+	1.5	27.65	31.26
12.	4^th^	+	+	+	3.74	36.06	39.90
13.	5^th^	+	+	+	4.32	42.68	48.72
14.	6^th^	+	+	+	4.48	50.67	58.53

The ‘+’ sign indicates the presence of *U. virens* and the ‘−’ sign indicates an absence of *U. virens* in the samples collected from the spore trap.

### DNA extraction and PCR

DNA was extracted according to the method outlined by Quesada et al. (2018). The air particles adhered to the silicone grease of the sampling rods were scraped off using a sterilized toothpick. The scraped material was transferred to a screw-capped vial containing 5 ml of cetyltrimethylammonium bromide (CTAB) buffer. The vial was vortexed, and 0.5 ml of this mixture was transferred to a 1.5 ml centrifuge tube. The tube was incubated at 65°C for 20 min. Subsequently, an equal volume of phenol, chloroform, and isoamyl alcohol (25:24:1) was added, followed by centrifugation at 12,000 rpm for 10 min. The resulting supernatant was collected, and 600 μl of ice-cold isopropanol was added. This mixture was then incubated overnight at −20°C. The sample was centrifuged after 24 h at 10,000 rpm for 10 min, followed by an ethanol wash. The supernatant was discarded, and the remaining pellet was resuspended in 50 μl of TE buffer for storage.

The extracted DNA was then subjected to a conventional PCR check using species-specific primers US1 and US3 (Table 2) to identify the presence of the target species, following the conditions mentioned by Zhou et al. (2003).

**Table 2:** Primers used in this study.

Primer	Sequence	Reference
US1	CCGGAGGATACAACCAAAAAAACTCT	Zhou et al. (2003)
US3	GCTCCAAGTGCGAGGATAACTGAAT	
FIP	GACAAGGGGGGGAACCGTTGTTGACATCAGGGCAGACAGA	Chandrasekar (2022)
BIP	ACAATGTCACTCCCCTGCAGTCCTGACGTGGCAACAGAGG	
F3	CACTGGTTCGGACGATGC	
B3	AGCAAACAATAAGCGACCCG	

FIP, forward inner primer; BIP, backward inner primer; F3, forward outer primer; and B3, backward outer primer.

### LAMP assay

The LAMP reactions mixture was made using 25 μl of LAMP reagents containing 2.5 μl of extracted sample DNA, 2.5 μl of Thermopol buffer (10X), 1.4 mM of each dNTPs, 0.3 mM of both outer primers, 1.5 mM of both inner primers (Table 2), 1.2 M Betaine, 8 mM MgSo_4,_ 2 U Bst DNA Polymerase (0.08 U/μl), and 120 μM of Hydroxy napthol blue (HNB) dye (Logeshwari et al., 2022). The total reaction mixture, except sample DNA, served as a control. The reaction was performed in an Eppendorf thermal cycler for 60 min at a constant temperature of 65°C. Then, the reaction was halted by thermal denaturation at 80°C for 2 min.

The colorimetric observation was conducted after reaction termination, in which the sky-blue color indicated a positive reaction and the purple color indicated a negative reaction. The quality of amplification was evaluated using 2% gel electrophoresis. The presence of a ladder-like pattern indicated the positive amplification.

### In-field detection of airborne inocula and development of spray schedule

The sampling rods were collected at weekly intervals from transplanting to harvest of 45th Standard Meteorological Week (SMW) to 6th SMW of 2023–2024, respectively. The DNA was extracted from each sample rod, as described earlier. The presence of airborne inocula was detected using the LAMP assay (2.4). The field trial was conducted using a randomized block design (RBD) with three treatments and seven replications to evaluate the effectiveness of an inoculum-based fungicidal spray schedule compared to the farmer’s practice. The broad-spectrum fungicides Azoxystrobin 16.7% + Tricyclazole 33.3% @ 0.1% concentration were used. Detailed information on treatment is given in Table 3. To evaluate the relationship between meteorological parameters and false smut disease incidence, field data on both environmental conditions and disease incidence were collected during the cropping season. The weather data were obtained from the automatic weather station (Yuktix Technologies, Bangalore, Karnataka) located at Paddy Breeding Station Coimbatore. The parameters such as maximum temperature (°C), minimum temperature (°C), relative humidity morning (%), relative humidity evening (%), and wind speed were recorded during the cropping season (45th to 6th SMW of 2023–2024). The disease incidence was determined by calculating the percent disease incidence (PDI) based on visual field observations of false smut symptoms on infected rice plants. Weekly disease incidence was recorded for different treatments (T1, T2, and T3). The disease incidence was determined according to Baite et al. (2020) using the following formula:


$$PDI=\;\frac{\mathrm{Number of infected tillers}}{\mathrm{Total number of tillers}}\;\times 100$$

**Table 3:** Treatment details on fixing the spray schedule for managing *U. virens* based on airborne inocula.

Treatments	Details
T_1_	Inoculum *cum* weather-based application of azoxystrobin 16.7% + tricyclazole 33.3% SC @ 500 ml/ha single spray
T_2_	Farmers practice (Curative application of azoxystrobin 16.7% + tricyclazole 33.3% SC @ 500 ml/ha) three sprays at a 15-day interval
T_3_	Untreated control

### Correlation analysis

A Pearson correlation analysis was conducted to assess the relationship between meteorological parameters and disease incidence. Pearson’s correlation coefficient (r) was used to measure the strength and direction of the linear relationships between disease incidence (a dependent variable) and each of the meteorological parameters (independent variables). The correlation analysis was performed using statistical software R (version 4.3.1) with the cor function. The weekly meteorological data (relative humidity, wind speed, temperature, sunshine, and solar radiation) were paired with the corresponding disease incidence data for each week (W45–W06). The results of the correlation were interpreted based on r values: (+1) indicates perfect positive correlation (as one variable increases, the other increases), (−1) indicates perfect negative correlation (as one variable increases, the other decreases), and 0 indicates no correlation.

**Figure 2:**
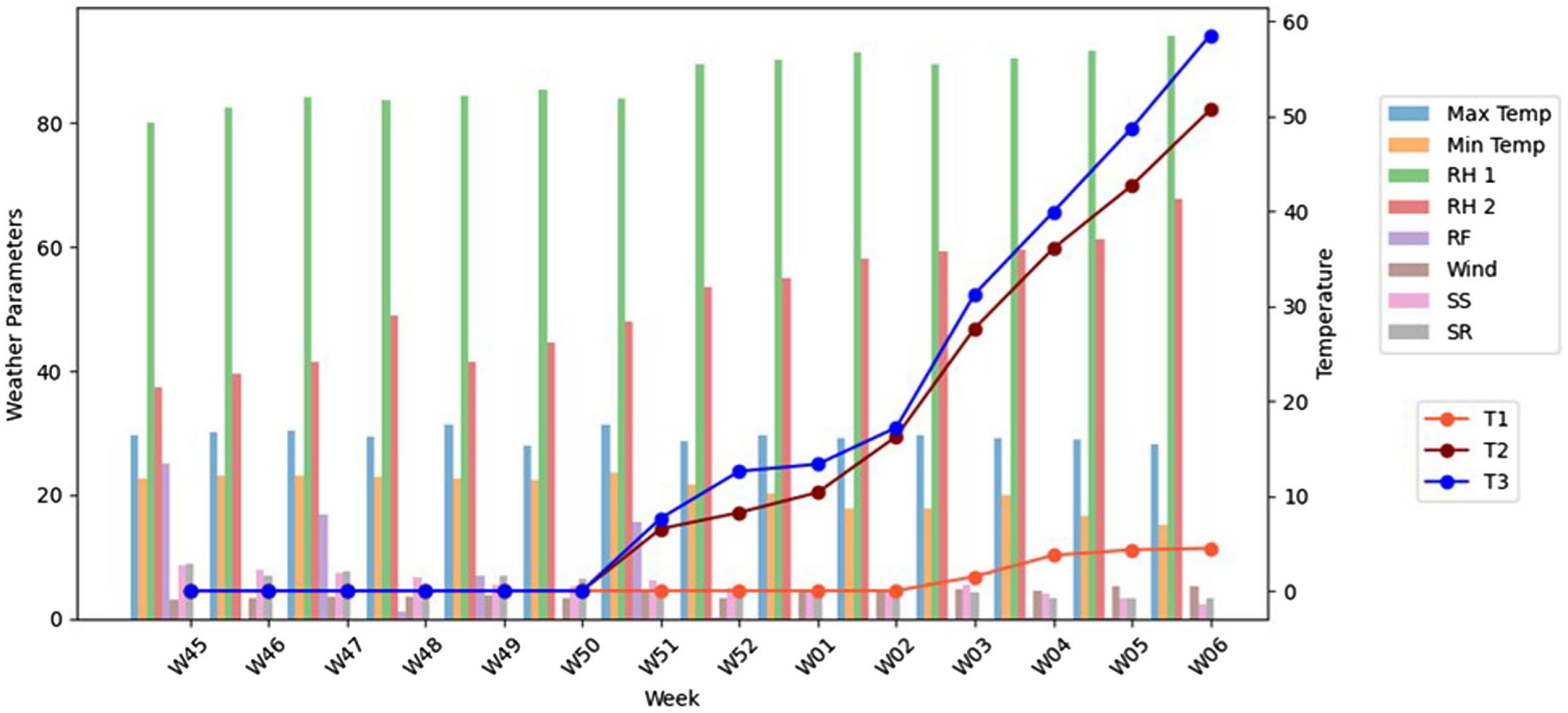
The graphical representation of three different treatments associated with weather parameters. The different colour bars represent each weather parameter (Maximum Temperatre, Minimum Temperature, Relative Humidity morning, Relative Humidity evening, Rain fall, Wind speed, Sunshine, Solar Radiation). The line graph represents treatment 1, 2, and 3.

A graphical representation (Figure 2) showed the trends of meteorological parameters and disease incidence over time. Weekly trends were plotted using ggplot2 in R, where disease incidence was compared against key weather variables. This approach allowed for a comprehensive understanding of how weather conditions influenced the progression of false smut disease in the field over time.

### Assessing the sensitivity of the LAMP assay

**Figure 3:**
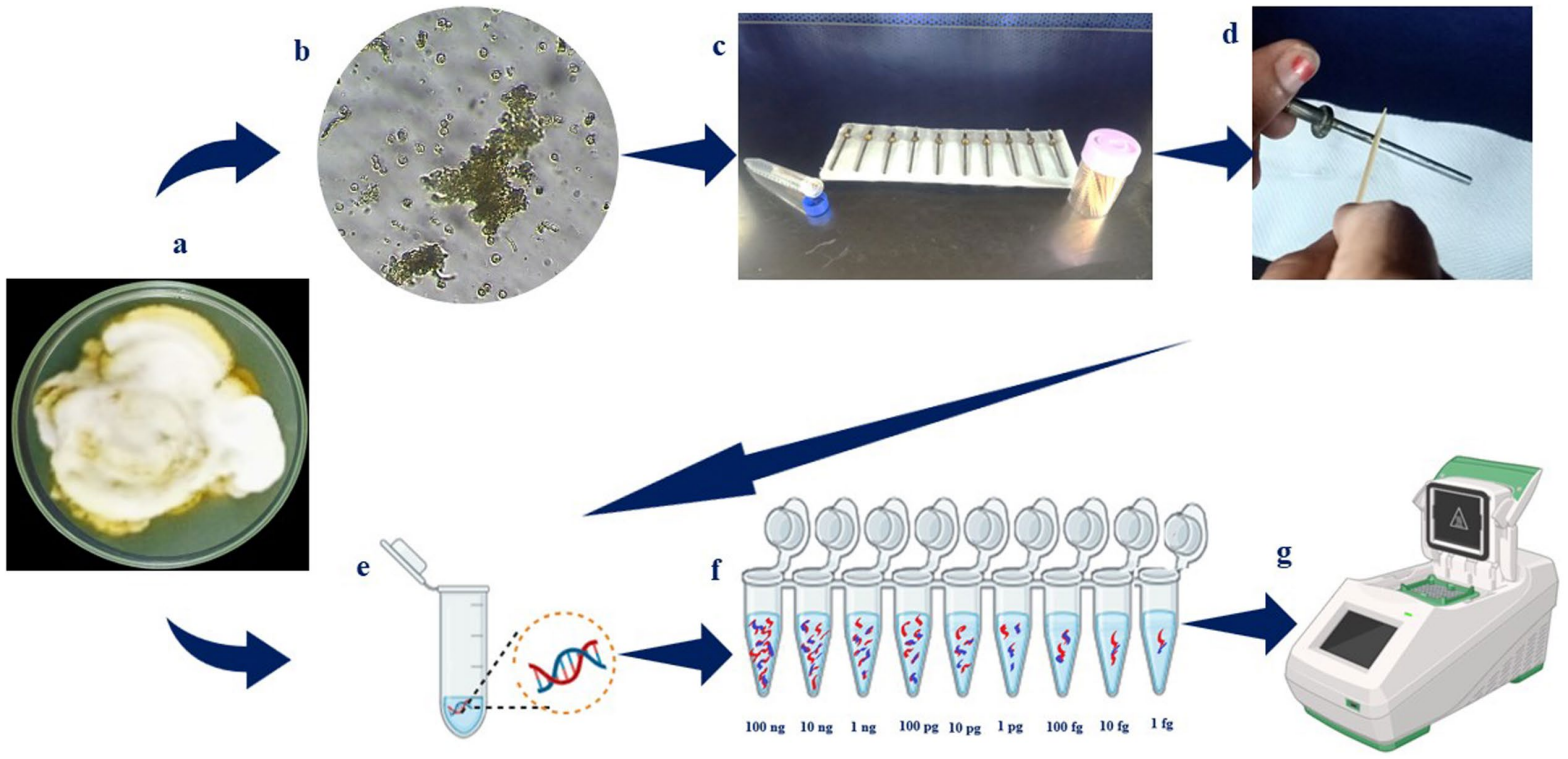
Overall flow on detection of *Ustilaginoidea virens*. (a) *Ustilaginoidea virens* culture grown on PSA media; (b) microscopic observation of U. virens spores; (c) sampling rods; (d) silicone grease coated over sampling rod; (e) the spore inocula added to rods in different dilution and DNA extracted; (f) as well as the DNA was extracted from the mycelial mat and diluted serially (100 ng, 10 ng, 1 ng, 100 pg,10 pg, 1 pg, 100 fg, 10 fg, 1 fg); (g) the samples were then subjected to PCR and LAMP assay.

To determine the lowest limit of detection using the LAMP assay, the purified genomic DNA was quantified using Nano-Drop One (Thermo Fisher Scientific, Waltham, MA, United States; Figure 3). Ten-fold serial dilutions were prepared from 10 ng to 1 fg of genomic DNA in nuclease-free water. One microliter of DNA from each dilution was added to the individual LAMP reaction mixture. The LAMP assay was performed following the same conditions and components described above. The DNA extracted from the conidial suspension spiked sampling rods was also detected through LAMP by adding 1 μl of DNA to the reaction mixture from each spiked sample. A negative control was included in each run.

### Culture collection and spike sensitivity of the sampling rods

Smut ball samples were collected from the infected plant samples at the paddy breeding station Coimbatore, Tamil Nadu, in 2023. To isolate the pure culture, the smut balls were surface-sterilized with 0.1% sodium hypochlorite and rinsed three times with sterile water. The smut balls were then dried on sterilized filter paper. Using a sterilized needle, the chlamydospores were streaked on the potato sucrose agar plate amended with streptomycin, per the protocol followed by Ladhalakshmi et al. (2012). The plates were incubated at 27°C for 7 days. The pure culture was obtained by repeated subculturing. The 100 ml milky grain broth (165 ml milky grain extract, made up to 1,000 ml with sterilized water) was inoculated with the agar plug from the pure culture, and it was incubated for 8 days at 25°C and 120 rpm. After that, 100 ml of potato sucrose broth (PSB) was added, and the mixture was once more incubated under the same conditions for a further week. Following this step, a cheesecloth was used to filter the culture, and then 0.05% Tween 20 (Sigma-Aldrich, St. Louis, MO, USA) was added to the suspension. The conidial suspensions were then pipetted onto rod sets placed in the vial.

To assess the sensitivity of the LAMP assay, the sampling rods were then spiked with a known concentration of serially diluted spore suspensions of *U. virens*. The conidial concentration was measured using a hemocytometer. Initially, the concentration was approxiamtely 1 × 10^4^ conidia/ml. It was then 10-fold serially diluted to 1 × 10^3^, 1 × 10^2^, 1 × 10, and 1 conidia and spiked to the sampling rod, respectively; the rod spiked with 0 conidia (only nuclease-free water) served as a control (Villari et al., 2017). Before further processing, the rods were allowed to air dry under laboratory conditions (28 ± 2°C) for 24 h (Figure 3).

## Results

### Monitoring dispersal of *U. virens* using spore trap

*U. virens* conidia were detected using LAMP assay from the sample collected from an air sampler (spore trap) placed in the trial plot during the cropping period. The spore dispersal was recorded at weekly intervals from the 45th to the 6th SMW of 2023–24 by collecting sampling rods from the spore trap. The microscopic observation of mounts prepared from sample rods revealed the presence of *U. virens*, *Bipolaris oryzae*, *Pyricularia oryzae*, *Alternaria*, and rice pollens (Supplementary Figure S1). Initially, the *U. virens* spores were not seen under a microscope. The spores were detected only at 48th SMW both under the microscope and LAMP assay, but in conventional PCR, it is detected only a week before visible symptom expression, i.e., on 50th SMW (Supplementary Figures S2, S3). This implies the higher sensitivity of the LAMP assay compared to conventional PCR to detect the lowest possible inoculum of *U. virens*. The first visible symptoms were seen only after 3 weeks from the detection of airborne inocula through the microscope, mainly observed at the panicle exertion stage 51^st^ SMW. The presence and absence of *U. virens* was confirmed primarily by microscopic observation. *Chlamydospores* were mostly seen under microspores in the sample collected from the spore trap. Further confirmation was achieved by the development of sky-blue color and ladder-like pattern formation under 2% agarose gel electrophoresis.

### Development of a spray schedule based on airborne inocula

The spray schedule was developed by inoculum-based spraying of azoxystrobin and tricyclazole as a single spray @ 0.1% at 50th SMW after booting and before flowering was recorded, with the minimum incidence of false smut (4.48%) with a maximum yield of 6,500 kg/ha. On the other hand, blanket application of fungicides based on farmers’ practices recorded a maximum of 50.67% of false smut incidence with a yield of 3,500 kg/ha. In the untreated control, the incidence of false smut reached the maximum of 58.53% and recorded a minimum yield of 1,500 kg/ha (Table 1; Figure 2). Similarly, an association was found between the frequency of disease and the meteorological parameters that dominated during the cropping season. Specifically, disease incidence increased with relative humidity and wind speed and decreased with temperature, sunshine, and solar radiation (Supplementary Tables S1, S2).

### Limit of detection and spiked assays using PCR

**Figure 4:**
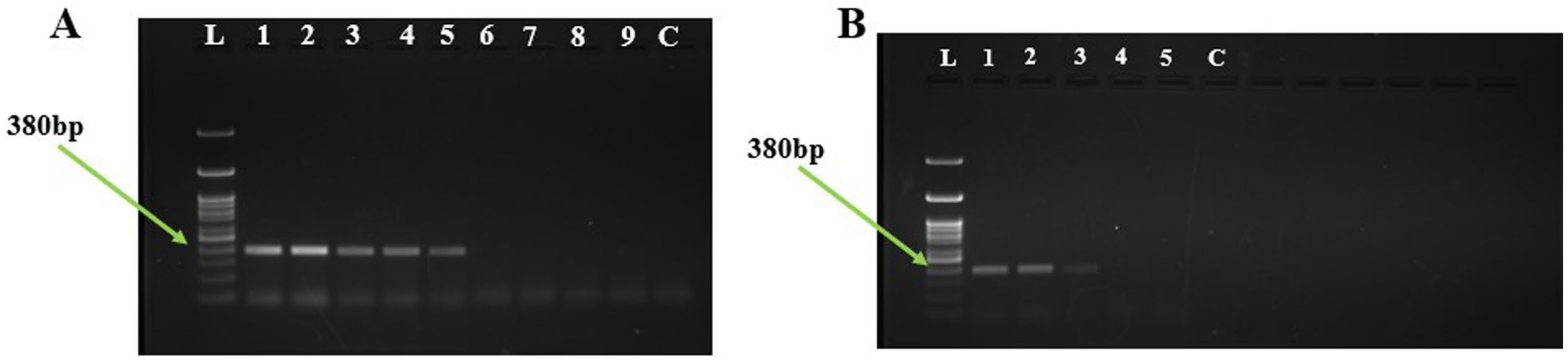
Detection of *Ustilaginoidea virens* using PCR assay. **(A)** Agarose gel electrophoresis analysis of the PCR products, each Lane represents L:1Kb Ladder; 1-100 ng; 2-10 ng; 3-1 ng; 4-100 pg; 5-10 pg; 6-1 pg; 7-100 fg; 8-10 fg; 9-1 fg; C-NFW (Negative control). **(B)** Agarose gel electrophoresis analysis of the PCR products, each Lane represents L:1Kb Ladder; 1–1 × 10^4^ conidia; 2-1 × 10^3^ conidia; 3-1 × 10^2^ conidia; 4-1 × 10 conidia; 5-1 conidia; C-NFW (Negative control). ng, nano gram; pg, pico gram; fg, femtogram; NFW, nuclease free water.

The limit of detection of the *U. virens* using PCR assay was determined by performing the assay on a tenfold serially diluted purified genomic DNA. The PCR reaction detected the target DNA at a minimum concentration of 10 pg (Figure 4). A total of six independent spore dilution series were used by adding 2.5 μl of DNA derived from sampling rods spiked with 0 to 1 × 10^4^ conidial spores. The PCR assay was able to amplify up to 1 × 10^2^ conidia at 380 bp, while it failed to amplify 1 and 10 conidia per sampling rod.

### Limit of detection and spiked assays using LAMP

**Figure 5:**
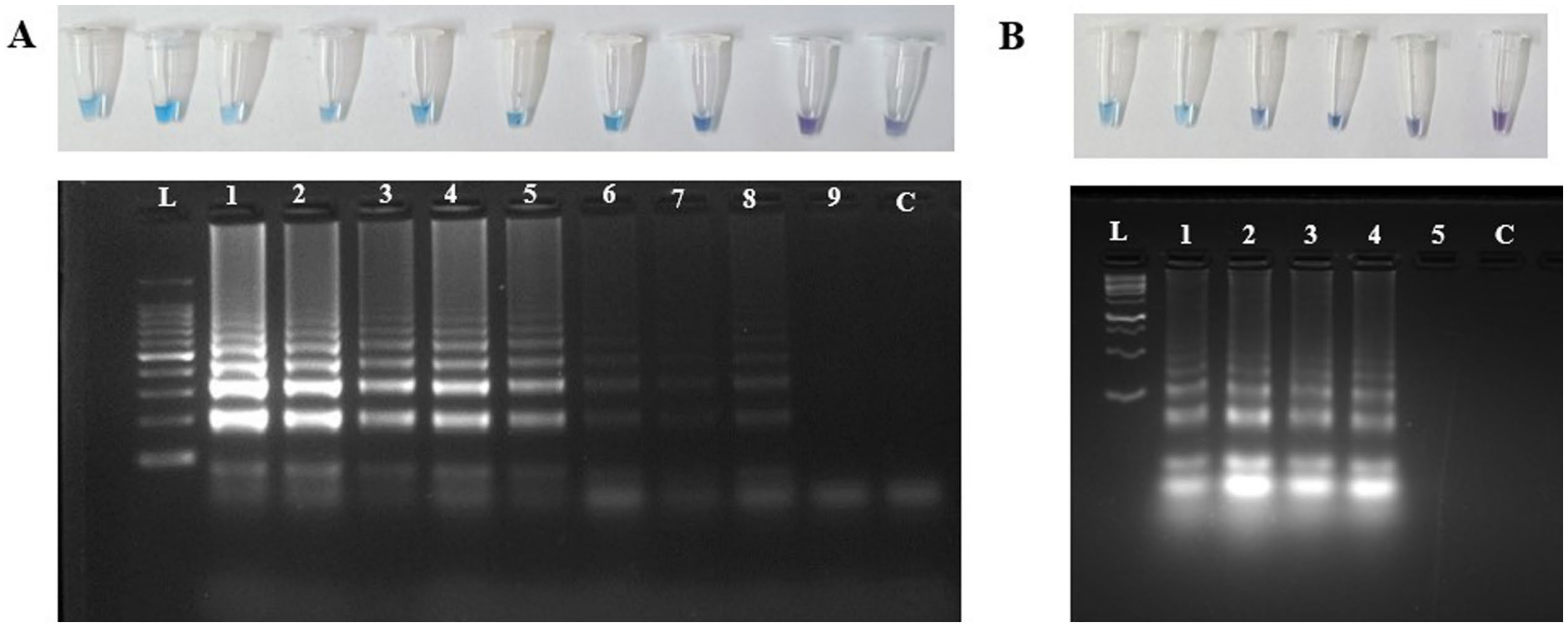
Detection of *U. virens* using LAMP assay. **(A)** Agarose gel electrophoresis analysis of the LAMP products, each Lane represents L:1Kb Ladder; 1-100 ng; 2-10 ng; 3-1 ng; 4-100 pg; 5-10 pg; 6-1 pg; 7-100 fg; 8-10 fg; 9-1 fg; C-NFW (Negative control). **(B)** Agarose gel electrophoresis analysis of the LAMP products, each Lane represents L:1Kb Ladder; 1-1 × 104 conidia; 2-1 × 103 conidia; 3-1 × 102 conidia; 4-1 × 10 conidia; 5-1 conidia; C-NFW (Negative control). ng, nano gram; pg, pico gram; fg, femtogram; NFW, nuclease free water.

The limit of detection of the *U. virens* LAMP assay was determined by performing the assay on a tenfold serially diluted preparation of purified genomic DNA. The results revealed strong sky-blue color development in all tubes containing different DNA concentrations except those containing 10 and 1 fg of DNA. Similarly, the ladder-like pattern was seen at a minimum concentration of 100 fg under 2% agarose gel electrophoresis (Figure 5). The LAMP amplified positive for all the serially diluted conidial spiking except for single conidia. The lowest possible detection of LAMP through spiking assay was about 1 × 10 conidia per sampling rod.

### Multi-operator validation and specificity

Three operators performed the LAMP assay with blind samples, including *U. virens* and other species, and a non-template control (Table 4). As a result, all three operators correctly identified *U. virens,* and there was no cross-reactivity with any other non-target samples.

**Table 4:** Multi-operator validation of loop-mediated isothermal (LAMP) assay specific for *U. virens.*

Crop	Isolate	LAMP test		
		Operator 1	Operator 2	Operator 3
Rice	*Rhizoctonia solani*	−	−	−
Rice	*Pyricularia Oryza*	−	−	−
Rice	*Bipolaris Oryza*	−	−	−
Rice	*Ustiloginoidea virens*	+	+	+
Tomato	*Alternaria solani*	−	−	−
Tomato	*Fusarium solani*	−	−	−

## Discussion

The management of plant diseases in the field primarily depends on reliable, efficient, and rapid detection methods. These methods must be affordable, field-compatible, and applicable to laboratories without sophisticated equipment. They should also be sensitive, specific, and able to detect various diseases (Yang et al., 2018). Thus, tracking airborne inocula of *U. virens* using spore traps, combined with Loop-Mediated Isothermal Amplification (LAMP) assays, provided a comprehensive surveillance method. Continuous monitoring of *U. virens* dispersal throughout the cropping season offered insights into the airborne dynamics of the pathogen. The inocula were detected 1–2 weeks before visible symptoms appeared on the rice panicles, offering a predictive clue for disease onset. Additionally, conidia from various pathogens were detected during the study, providing real-time data crucial for predicting disease outbreaks and implementing timely management strategies.

Spore trap samples were initially analyzed using microscopy (Kim et al., 2019). However, this method was labor-intensive, and identifying *Diplocarpon coronariae* spores in field samples proved difficult due to the complexity of the samples (Boutry et al., 2023). Although quantitative PCR (qPCR) is another sensitive and specific method capable of quantifying as few as 10 conidia per sample, it shares some limitations: being labor-intensive and not cost-effective, similar to other qPCR techniques used for fungi (Calderon et al., 2002; Dvorák et al., 2015; Luchi et al., 2013). In contrast, the LAMP assay offers several advantages. It is labor-friendly, cost-effective, and highly specific when targeting conserved fungal sequences. When combined with spore trapping, it enables more effective disease management. For example, a LAMP assay targeting the cyt b region of *Uromyces betae* was developed by Kaczmarek et al. (2019). This assay, when coupled with spore traps in sugar beet fields, demonstrated timely pathogen detection, enabling effective disease control.

The coupling of LAMP with spore traps also helped reveal the spatiotemporal pattern of inoculum presence and was used to develop inoculum-based forewarning models for conidia detection. This proactive approach to managing false smut disease, by detecting airborne *U. virens* inocula, proved more effective than traditional methods. Detecting *U. virens* inocula before visible symptoms allowed fungicide application at an optimal time before or at infection onset. This early intervention minimized disease progression, as observed in the field trial, where the inoculum-based schedule (T1) resulted in a lower false smut incidence (4.48%) and a higher yield (6,500 kg/ha). In contrast, the farmer’s practice (T2) had a significantly higher disease incidence (50.67%) and a lower yield (3,500 kg/ha), while the untreated control (T3) showed the highest disease incidence (58.53%) and the lowest yield (1,500 kg/ha).

By reducing the need for multiple fungicide applications, this inoculum-based approach also lowered costs and minimized environmental impacts, aligning with sustainable agricultural practices. Additionally, targeted fungicide use reduces the risk of fungicide resistance development, preserving their effectiveness. Environmental factors such as humidity and wind speed were found to correlate positively with disease incidence, while higher temperatures and more sunshine reduced it. By combining inoculum detection with weather forecasting, farmers can further refine management strategies and apply fungicides when most needed.

Standardizing fungicide applications based on airborne inocula detection using spore traps reduced the number of sprays, minimized crop damage and yielded economic benefits across several crops, including *Botrytis cinerea* in grapevine, *Cercospora sojina* in soybean, *Fusarium graminearum* in wheat and *Bremia lactucae* in lettuce (González-Fernández et al., 2020; Zuchelli, 2021; Hellin et al., 2018; Kunjeti et al., 2016; Carisse et al., 2012). The comprehensive statistical analysis highlighted the correlation between weather parameters and disease incidence. Increased relative humidity and wind speed were positively associated with disease occurrence, whereas low minimum temperatures, solar radiation, and sunshine resulted in a higher incidence of disease. This relationship was further validated by findings in West Bengal, where both maximum and minimum temperatures, relative humidity, and wind speed were critical for false smut development, whereas, in Gujarat, maximum temperature and relative humidity alone were the main contributors (Saha et al., 2020; Chaudhari et al., 2019).

The LAMP assay demonstrated high sensitivity, detecting *U. virens* DNA at concentrations as low as 100 fg, significantly outperforming conventional PCR, which could detect only up to 100 pg (Figure 4). The rapid detection of *Alternaria solani* using LAMP yielded more reliable and accurate results, with amplification occurring in less than 60 min at 63°C, showing a 10-fold greater sensitivity than conventional PCR (Khan et al., 2018). This high level of sensitivity ensures that the disease can be detected early, providing sufficient time for timely interventions.

The robustness and reproducibility of the LAMP assay were confirmed through validation by three different operators, with no cross-reactivity observed in non-target samples. The spiked assays further demonstrated the assay’s capability, detecting as few as 1 × 10 conidia per rod, compared to conventional PCR’s detection limit of more than 1 × 10^2^ conidia (Figure 3). The multi-primer system of the LAMP assay (using inner and outer primers) enhanced both specificity and amplification speed, contributing to its superior performance in detecting small amounts of *U. virens* DNA compared to conventional PCR. This sensitivity level enables early inoculum detection in the air at low concentrations, which is crucial for preemptive disease management.

In conclusion, this study presents a novel and integrated approach for managing false smut disease in rice by combining spore trapping technology for airborne pathogen surveillance with the LAMP assay for rapid and sensitive detection of *U. virens*. This approach offers significant potential for improving the sustainability of rice production and enhancing food security, particularly in regions vulnerable to airborne diseases. Further research and field trials are needed to validate these findings across rice-growing regions and cropping systems.

## Data Availability

The original contributions presented in the study are included in the article/Supplementary material, further inquiries can be directed to the corresponding author.
