# Supplementary material for: Integrating spore trapping technology with loop-mediated isothermal amplification assay for surveillance and sustainable management of rice false smut disease
Source: Front Microbiol. 2024 Dec 4;15:1485275. doi: 10.3389/fmicb.2024.1485275 (PMC11652660; doi:10.3389/fmicb.2024.1485275)
Supplement: Supplementary file 1 [file Data_Sheet_1.DOCX]

Supplementary Material


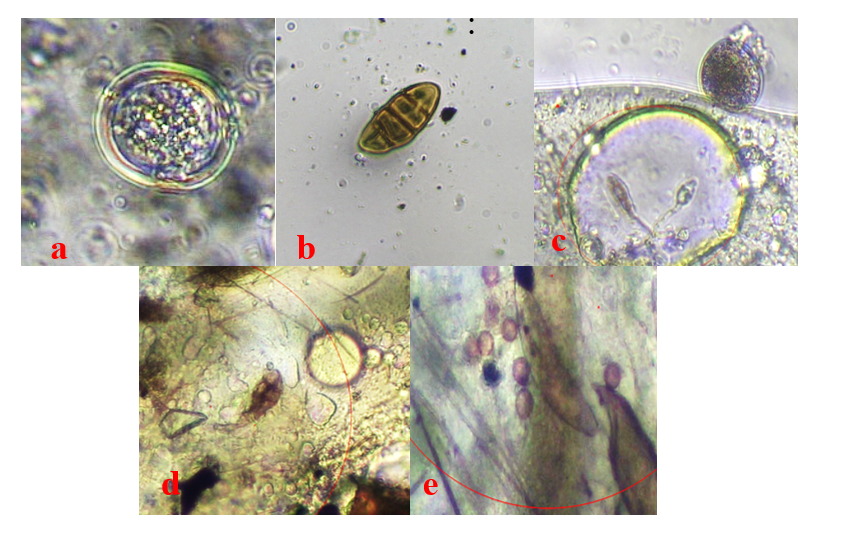


**Figure S1: Microscopic observations of various fungal spores and particles collected from spore traps** a) Ustilaginoidea virens chlamydospore*,* b) Bipolaris oryzae conidium*,* c) Pyricularia oryzae conidium*,* d) Alternaria species conidia and e) Rice pollen grains.

**
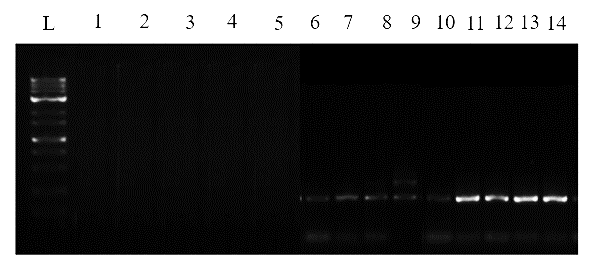
**

**Figure S2: Detection of *U. virens* using spore trap** (A) Agarose gel electrophoresis analysis of the spore trap samples detected through PCR assay, each Lane represents L:1Kb Ladder; 1- 45^th^ SMW; 2- 46^th^ SMW; 3- 47^th^ SMW; 4- 48^th^ SMW; 5- 49^th^ SMW; 6- 50^th^ SMW; 7- 51^st^ SMW; 8- 52^nd^ SMW; 9-1^st^ SMW; 10- 2^nd^ SMW; 11- 3^rd^ SMW; 12- 4^th^ SMW; 13- 5^th^ SMW; 14- 6^th^ SMW

**
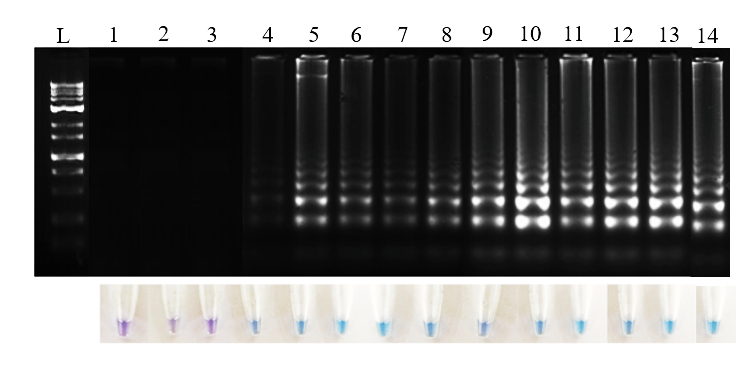
**

**Figure S3: Detection of *U. virens* using spore trap** (A) Agarose gel electrophoresis analysis of the spore trap samples detected through LAMP assay, each Lane represents L:1Kb Ladder; 1- 45^th^ SMW; 2- 46^th^ SMW; 3- 47^th^ SMW; 4- 48^th^ SMW; 5- 49^th^ SMW; 6- 50^th^ SMW; 7- 51^st^ SMW; 8- 52^nd^ SMW; 9-1^st^ SMW; 10- 2^nd^ SMW; 11- 3^rd^ SMW; 12- 4^th^ SMW; 13- 5^th^ SMW; 14- 6^th^ SMW.

**Table S1: Weather and disease incidence data from SMW 45- SMW 6:**

| **SMW** | **Max** | **Min** | **RH 1** | **RH 2** | **RF** | **Wind** | **SS** | **SR** | **T1** | **T2** | **T3** |
| --- | --- | --- | --- | --- | --- | --- | --- | --- | --- | --- | --- |
| W45 | 29.70 | 22.71 | 80.00 | 37.43 | 25.14 | 2.97 | 8.63 | 8.81 | 0 | **0** | **0** |
| W46 | 30.09 | 23.14 | 82.57 | 39.57 | 0.00 | 3.26 | 8.00 | 7.00 | 0 | 0 | 0 |
| W47 | 30.37 | 23.17 | 84.23 | 41.43 | 16.74 | 3.51 | 7.44 | 7.76 | 0 | 0 | 0 |
| W48 | 29.33 | 22.86 | 83.65 | 49.00 | 1.16 | 3.61 | 6.56 | 5.26 | 0 | 0 | 0 |
| W49 | 31.21 | 22.54 | 84.54 | 41.57 | 7.00 | 3.77 | 5.46 | 6.89 | 0 | 0 | 0 |
| W50 | 28.00 | 22.33 | 85.43 | 44.71 | 0.00 | 3.36 | 5.34 | 6.47 | 0 | 0 | 0 |
| W51 | 31.43 | 23.60 | 83.86 | 48.00 | 15.60 | 4.34 | 6.17 | 5.30 | 0 | 6.53 | 7.64 |
| W52 | 28.73 | 21.60 | 89.50 | 53.63 | 0.06 | 3.25 | 5.08 | 4.29 | 0 | 8.24 | 12.59 |
| W01 | 29.61 | 20.30 | 90.14 | 55.00 | 0.00 | 4.20 | 4.71 | 4.34 | 0 | 10.36 | 13.34 |
| W02 | 29.21 | 17.81 | 91.43 | 58.14 | 0.00 | 4.86 | 4.39 | 4.90 | 0 | 16.25 | 17.19 |
| W03 | 29.74 | 17.70 | 89.43 | 59.29 | 0.00 | 4.66 | 5.37 | 4.14 | 1.5 | 27.65 | 31.26 |
| W04 | 29.21 | 19.96 | 90.43 | 59.57 | 0.07 | 4.53 | 3.97 | 3.26 | 3.74 | 36.06 | 39.9 |
| W05 | 28.93 | 16.50 | 91.71 | 61.29 | 0.21 | 5.19 | 3.39 | 3.27 | 4.32 | 42.68 | 48.72 |
| W06 | 28.29 | 15.21 | 94.00 | 67.71 | 0.00 | 5.31 | 2.44 | 3.19 | 4.48 | 50.67 | 58.53 |

**Table S2: Correlation analysis of weather parameter and disease incidence**

|  | *Max* | *Min* | *RH 1* | *RH 2* | *RF* | *Wind* | *SS* | *SR* | *PDI-T3* |
| --- | --- | --- | --- | --- | --- | --- | --- | --- | --- |
| Max | 1 |  |  |  |  |  |  |  |  |
| Min | 0.51726 | 1 |  |  |  |  |  |  |  |
| RH 1 | -0.5256 | -0.88197 | 1 |  |  |  |  |  |  |
| RH 2 | -0.49993 | -0.89316 | 0.946933 | 1 |  |  |  |  |  |
| RF | 0.538062 | 0.508264 | -0.67204 | -0.616 | 1 |  |  |  |  |
| Wind | -0.17405 | -0.86204 | 0.812213 | 0.871217 | -0.41306 | 1 |  |  |  |
| SS | 0.488677 | 0.80964 | -0.93285 | -0.89812 | 0.649174 | -0.81778 | 1 |  |  |
| SR | 0.41898 | 0.736714 | -0.88391 | -0.94372 | 0.710832 | -0.78468 | 0.874226 | 1 |  |
| PDI-T3 | -0.44395 | -0.90425 | 0.841719 | 0.902519 | -0.43444 | 0.857415 | -0.81983 | -0.81916 | 1 |

r = -1 to +1, Max = Maximum temperature, Min = Minimum Temperature, RH1 = Relative Humidity (morning), RH2 = Relative Humidity (evening), RF = Rainfall, Wind = Windspeed, SS = Sunshine, SR = Solar Radiation, PDI = Percent Disease Incidence.
